# Barriers to Acceptance of Provider-Initiated Testing and Counseling among Men Who Have Sex with Men in Shenyang, China: A Cross-Sectional Study

**DOI:** 10.1155/2013/280969

**Published:** 2013-07-01

**Authors:** Qinghai Hu, Junjie Xu, Zhenxing Chu, Jing Zhang, Ke Yun, Feng Shi, Yongjun Jiang, Wenqing Geng, Hong Shang

**Affiliations:** Key Laboratory of AIDS Immunology of the Ministry of Health, Department of Laboratory Medicine, No. 1 Hospital of China Medical University, 155 Nanjing North Street, Shenyang 110001, China

## Abstract

A high prevalence of HIV infection is present among men who have sex with men (MSM) in China, but many people living with HIV or AIDS (PLWHs) are unaware of their HIV infection status. Provider-initiated HIV testing and counseling (PITC) is a streamlined model that can significantly enhance HIV detection and detect infections earlier. However, PITC has not yet been widely applied, and no studies have been conducted on MSM's attitudes towards PITC in China. In this study, a total of 438 MSM were recruited in Shenyang city. A multivariate logistic regression model showed that certain conditions made MSM more accepting of PITC: those who had attended VCT (voluntary counseling and testing) more than three times (odds ratio [OR]: 2.95, 95% CI: 1.36–6.37), those who considered PITC beneficial for family and friends (OR: 1.91, 95% CI: 1.25–2.92), those who obtained HIV/AIDS knowledge from brochures (OR: 2.52, 95% CI: 1.64–3.87), those who obtained HIV/AIDS knowledge from the Internet (OR: 1.66, 95% CI: 1.07–2.58), and those who were highly aware of their own risk of being infected with HIV (OR: 2.84, 95% CI: 1.37–5.91). To improve acceptance of PITC among MSM in China, stronger efforts are needed to lower the psychosocial barriers to receiving PITC, to promote HIV/AIDS awareness, and to encourage the extension of HIV testing.

## 1. Introduction 

Human immunodeficiency virus (HIV) testing is the first step in many HIV prevention, care, and treatment programs because testing allows individuals who are at high risk for HIV infection to learn about their infection status and access appropriate services [1, 2]. As antiretroviral therapy (ART) becomes more widely available in resource-limited settings, increasingly, the challenge is case identification so that patients may benefit from ART in a timely fashion and improve their quality of life. Moreover, HIV testing may also help to prevent HIV secondary transmission [3]. Data from across China show there are an estimated 780,000 people living with HIV/AIDS (PLWHs) in 2011, and, unfortunately, more than 56% of PLWHs are unaware they have been infected [4]. Identifying these people and the status of their infection has never been more important and urgent. There are currently an estimated 18 million men engaging in homosexual sex in China [5]. More importantly, HIV transmission among homosexuals rose from 0.3% before 2005 to more than 13.7% in 2011 [4, 5]. Thus, in addressing the China AIDS epidemic, it is critical to control the spread of HIV among MSM.

Voluntary counseling and testing (VCT) for HIV has been demonstrated to be one of the most effective strategies in identifying HIV-infected individuals [1, 6]. VCT was highly efficacious in reducing risky sexual risk behaviour, especially for high-risk populations [1, 7]. However, there are also barriers associated with VCT participation, which include clients having to initiate testing themselves, lengthy pre- and posttest counseling, and patient concerns about confidentiality [8, 9]. Because of the double discrimination they face because of their sexual orientation and being HIV-infected, Chinese MSM have great fear of being exposed. Thus, most Chinese MSM experience high affective barriers to proactively take part in VCT for HIV.

Provider-initiated HIV testing and counseling (PITC) is a streamlined model promoted by the World Health Organization (WHO) in 2007 [10]. WHO policy guidelines suggest that HIV testing and counseling has to be offered by health care providers as part of the routine care of all patients or clients [10–12]. Compared with VCT, PITC simplifies information acquisition and counseling about high-risk behaviour and allows MSM to avoid the “humiliation” and “inconvenience” they fear. In this way, PITC can increase HIV detection rates, catch HIV infections earlier, and, thus, improve cost-effectiveness by cutting down on treatment costs [10, 13, 14]. A study from The Netherlands found that a PITC strategy decreased the rates of MSM refusals to receive HIV testing from 38% in 2006 to 12% in 2007 [15]. Also because of PITC, the rate of HIV detection by hospitals increased from 10.7% in 2005 to 29.4% in 2008 in Yunnan Province (Lin Lu, unpublished data). Nearly 71.4% of those who were found to be HIV positive in the clinic of the First Affiliated Hospital of China Medical University self-identified as MSM (Hong Shang, unpublished data), which suggests that the PITC carried out in the hospital seemed to be especially beneficial among MSM and, possibly, other high-risk groups. Some studies from the USA and Uganda have shown that PITC has been adopted by between 53% and 95% of the emergency departments in these countries and has led to substantial increases in participation in HIV testing [16, 17]. However, the psychosocial factors associated with PITC acceptance have not been previously examined among Chinese MSM.

The primary aim of this study was to evaluate the acceptability of PITC among MSM in Shenyang, China. Shenyang is an economic, cultural, and industrial hub of Liaoning province. Shenyang has the second largest MSM population in China (after Beijing) with an estimated 140,000 MSM who are from the city [18]. Previous regional studies of Shenyang and its surrounding areas revealed both high HIV seroincidence [19] and high prevalence among local MSM [20], but there have been no studies examining MSM attitudes towards PITC. Exploring the willingness of Shenyang MSM to accept PITC will help inform intervention policies or strategies for MSM throughout China.

## 2. Materials and Methods

### 2.1. Study Design and Participant Enrolment

The study was conducted from June to September 2010, during which MSM were recruited through snowball sampling via a nongovernmental organization (NGO), the Shenyang Sunny Workgroup, which provides health education outreach to Shenyang MSM. Recruited individuals were interviewed at the HIV Voluntary Counseling and Testing Clinic at the First Affiliated Hospital of China Medical University. The inclusion criteria for participating in the study were male, HIV negative, at least 18 years of age, reported having had at least one male sexual partner with whom he had receptive and/or insertive anal sex within the past 12 months, and physically able and willing to provide written informed consent.

### 2.2. Ethics

Ethical approval was obtained from the Institutional Review Board of the First Affiliated Hospital of China Medical University prior to the commencement of the study. Permission to conduct the study was sought from the local administrative authority. Informed consent was obtained from all participants after they received an explanation of the study's aim. For confidentiality and privacy, unique identification numbers were used instead of names.

### 2.3. Data Collection

An anonymous questionnaire was designed with the primary aim of obtaining information about the subject's acceptance of PITC. Other demographic, cognitive, and behavioral variables were considered to ascertain their influence on the participant's willingness to accept PITC. The majority of the questions have been widely used or reported in other literature. The questionnaire consisted of 31 questions divided into three sections: (1) sociodemographic characteristics, (2) knowledge and attitudes concerning HIV/AIDS and PITC, and (3) sexual behavior. In the first section, participants were asked about basic demographic information, such as nationality, age, ethnicity, education level, job, monthly income, and preferred medium for seeking sexual partners. The second section was designed to determine the subject's willingness to accept PITC as well as knowledge regarding HIV prevention—such as transmission routes, prevention methods, and correct use of condoms—and their knowledge about the benefits of PITC. In section three, the questions dealt with sexual health and behavior. Interviews were administered face-to-face by trained physicians or volunteers from NGOs. Investigators introduced PITC to all participants before the interview. A small incentive equivalent to USD $5 was given to each participant as compensation for their time.

### 2.4. Data Analysis

Questionnaire data were entered twice and then checked for accuracy using Epi Data software (the Epi Data Association Odense, Denmark, version 3.02). Data were then analyzed using SAS 9.1 (SAS Institute Inc., Cary, NC, USA). Descriptive statistics and univariate analysis were generated for each of the variables corresponding to specific questions in the survey.

There were eleven questions related to HIV/AIDS prevention knowledge that participants were asked. Answering one question correctly earned them one point. Bivariate logistic analysis was performed to study the association of each of the independent variable with an expression of interest in PITC (which was considered acceptance of PITC). The majority of these independent variables were dichotomized based on the presence (a “Yes” response) or absence (a “No” response) of the specific characteristic being examined. Age was arbitrarily dichotomized as “older” and “younger” using the approximate median age (28 years) of participants as the cutoff, and high school-level education and 1000 Chinese Yuan (CNY) per month were used as cutoffs for the dichotomous variables “education” and “monthly income”, respectively. Contingency tables were constructed for all comparisons and the chi-square (*χ* ^2^) test; Mantel-Haenszel statistics and measures of association were calculated (Odd Ratios). Test statistics were considered significant if *P* values were less than 0.05, and 95% confidence intervals were calculated for the measures of association.

Backwards stepwise multivariate logistic regression analysis was performed to identify factors associated with the acceptance of PITC. Variables that were significant in univariate analysis (*P* < 0.20) were assessed for multicollinearity by using variance inflation factors and tolerance. Then, variables without multicollinearity were considered in multivariate analysis. Only variables with *P* < 0.05 were kept in the final multivariate logistic model in a stepwise manner.

## 3. Results

### 3.1. Demographic Characteristics of MSM Participants

A total of 441 MSM were interviewed in the study, but three of them were disqualified from participation because they were less than 18 years of age.  Table 1 provides an overall description of the demographic characteristics of the study population. The median age was 28 years with a range between 18 and 75 years of age. The majority of participants were ethnically Han (84.5%), and more than half possessed a high school-level education or higher (57.0%). For 47% of the participants (205), the Internet was their preferred medium for seeking sexual partners.

### 3.2. Behavioral Characteristics of MSM Participants

We found that 22.4% of the participants had engaged in sex with men, and 13.5% had engaged in sex with women before the age of 18. About 296 participants (67.6%) had had regular male sexual partners in the past 12 months, and only 24.7% (73/296) used condoms with these partners during every instance of sexual intercourse. In addition, 352 participants (80.4%) had casual male sexual partners in the past 12 months, and 28.7% of them (101/352) used condoms with these sexual partners during every instance of sexual intercourse. About 400 participants (91.3%) had previously been tested for HIV. The average scores of AIDS prevention knowledge were 8.8 points out of 11, and about 93 participants (21.2%) answered all questions correctly. There were 291 participants (66.4%) who obtained their knowledge from the Internet. Of all the participants, 266 (60.7%), 287 (65.5%), and 279 (63.7%) obtained their knowledge from brochures, friends, and televised public advertisements, respectively.

### 3.3. Willingness to Receive PITC

The number of people who identified themselves as being at high risk for HIV infection was 50 (11.4%); 259 (59.1%) self-identified as being at low risk for HIV infection; and 97 (22.2%) self-identified as being at no risk for HIV infection. After receiving an explanation about PITC, 53.7% of participants reported that they would be willing to receive PITC if the procedure was offered to them. The major reasons for declining PITC were the fear of violation of privacy/rights and self-identification as being at no risk or low risk for HIV infection.

Table 2 displays the factors that significantly correlate with the willingness to receive PITC. Correlations were drawn using univariate analysis in which certain factors were found to be significantly associated with the willingness to receive PITC (*P* < 0.05). These factors were self-identification of high risk for HIV infection; HIV/AIDS prevention knowledge scores; HIV testing frequency through VCT; seeing PITC as beneficial for family and friends; obtaining HIV/AIDS prevention knowledge from the radio; obtaining HIV/AIDS prevention knowledge from televised public advertisements; obtaining HIV/AIDS prevention knowledge from newspapers and magazines; obtaining HIV/AIDS prevention knowledge from friends; obtaining HIV/AIDS prevention knowledge from brochures; obtaining HIV/AIDS prevention knowledge from the Internet.

In the final adjusted multivariate model (Table 3), the strongest predictors of an MSM's acceptance of PITC were having been previously tested for HIV more than three times in a VCT clinic (OR = 2.95, 95% CI: 1.36–6.37, *P* = 0.006), considering PITC beneficial to family and friends (OR = 1.91, 95% CI: 1.25–2.92, *P* = 0.003), having obtained AIDS prevention knowledge from brochures (OR = 2.52, 95% CI: 1.64–3.87, *P* < 0.001), having obtained AIDS prevention knowledge from the Internet (OR = 1.66, 95% CI: 1.07–2.58, *P* = 0.025), and self-identification as being at high risk for HIV infection (OR = 2.84, 95% CI: 1.37–5.91, *P* = 0.005).

## 4. Discussion 

This study was the first to explore the acceptance of PITC among Shenyang MSM. Understanding various characteristics of this population is important for drawing conclusions that have plausible implication for the prevention and control of China's nationwide HIV/AIDS epidemic. In the present study, the proportion of MSM who had not previously heard of PITC was nearly 45%. After learning about PITC, 53.7% (235/438) of participants considered it necessary to implement PITC in hospitals. This percentage is slightly lower compared to attitudes reported in another study conducted on Chinese female sex workers (FSWs) in which the median acceptance of PITC was 59.8% [21]. These percentages suggest that the MSM subpopulation is just as important target as FSWs. Another study involving TB patients showed that their acceptance rate of PITC was 99.1% in Guangxi province, southern China [22]. Regarding resistance to PITC—for reasons that include the fear of receiving a positive test result or a self-evaluation of being at no or low risk for HIV infection—the proportion of MSM refusing PITC is higher than that of other high-risk populations [15]. Because Chinese society emphasizes the traditional obligation to uphold familial reputations and lineages, MSM are threatened by the stigma associated with the exposure of their sexual orientation in China [5, 23], resulting in a fear of seeking out HIV-related information, resources, and testing. Therefore, this study must be applied together with efforts to promote tolerance of sexual orientation and oppose discrimination. In summary, more health education and social intervention should target MSM, and more resources should be allocated to HIV prevention among MSM. 

Participants who declined PITC gave reasons such as fear of having their privacy or rights violated and identifying themselves as being at no or low risk for HIV infection. In the United States, “having no risk for HIV infection” is also a common reason for declining routine HIV testing [16, 24]. These findings confirm that perception of risk is one of the main reasons that participants declined testing for HIV. Our surveys demonstrated that people who self-identify as being at high risk for HIV infection were more likely to accept PITC (OR = 2.84, 95% CI: 1.37–5.91). This information strongly suggests that patients need to be educated about the actual risks and the realities of HIV infection. We also found that those who learned about AIDS prevention from brochures were more willing to accept PITC (OR = 2.52, 95% CI: 1.64–3.87). This finding implies that, when offering HIV testing, health care providers should provide educational printed materials about HIV risk factors. This information can help patients better understand their personal risks and may decrease the number of patients who decline testing based on their perception of their risk.

Also, our results highlight a visible relationship between being aware that HIV prevention is important and accepting PITC (OR = 0.024, 95% CI: 1.06–2.28). Although the scores from the HIV prevention knowledge portion of the questionnaire were not entered into the multivariate analysis model of PITC acceptance factors, our study found that only 21.2% (93/438) of participants answered all 11 HIV prevention questions correctly. Meanwhile, the rates of condom use with regular and casual sexual partners were both low (24.7% with regular sexual partners and 28.7% with casual sexual partners). Similarly, MSM participants from Beijing and Jinan had lower awareness of AIDS knowledge and lower rates of condom use [25, 26]. AIDS prevention knowledge informs attitudes about AIDS and the development of safe sexual habits. Thus, to enhance basic HIV/AIDS knowledge among MSM in Shenyang, targeted health education should be carried out and sustained. 

Furthermore, multivariate analysis found that MSM who had been tested for HIV at VCT clinics more than three times showed significantly higher acceptance of PITC compared to MSM who never received VCT (OR = 2.95, 95% CI: 1.36–6.37). This finding illustrates that VCT plays a critical role in MSM consultations, education, and intervention. Despite there being more than 4000 VCT sites in China in 2007, the average number of attendees was less than two per day [27, 28]. These numbers underscore the urgent need for greater efforts to increase VCT utilization in China. Only 19.1% of VCT clinics were located in hospitals in 2007 [27]. In 2009, 28.9% of hospitals reported having a VCT clinic according to data from Internet-based reporting of HIV/AIDS in China, compared with 25.0% in 2008 [29, 30]. Patients who had been tested for HIV in hospitals were tested as part of the routine examination of inpatients. This phenomenon implies that expanding HIV testing to all outpatients would greatly increase the number of people being tested. Because of the double stigma attached to being HIV positive and homosexual, MSM may avoid testing and treatment facilities to avoid the potential for discrimination [31]. Interestingly, many participants preferred accepting HIV testing in hospitals rather than in testing and treatment facilities [8]. HIV/AIDS patients can receive integrated services, such as referrals, intervention, treatment, and timely care, if they are diagnosed with HIV in a hospital. Thus, the government should continue its support of VCT for AIDS prevention, work on increasing PITC acceptance among MSM, and design methodologies for earlier HIV detection among MSM to reduce the risk of secondary HIV transmission.

Another important finding was that the Internet was the most common source (46.8%) for seeking male sex partners for the participants in our study. We also found that those learning about AIDS prevention from the Internet showed higher rates of PITC acceptance (OR = 1.66, 95% CI: 1.07–2.58). Studies have indicated that MSM who often used the Internet have more favourable attitudes toward online methods of promoting the prevention of STIs (sexually transmitted infections)/HIV [32]. Zhang's study showed that more than one-third of respondents reported using the Internet to research health information [33]. These findings show that the Internet is an effective means of providing HIV prevention and health education information to MSM and, therefore, should be part of forefront intervention strategies for PITC education.

We also found that MSM who believed PITC could benefit their family and friends displayed higher rates of PITC acceptance (OR = 1.91, 95% CI: 1.25–2.92). Due to social pressures and traditional family values, approximately 70–80% of Chinese MSM are currently, or will eventually get, married to a woman [34]. The influence of traditional Chinese culture causes Chinese people to see their families as their main source of support in cases of severe disease. This impact of culture on behaviour demonstrates that widespread family-based HIV prevention strategies that utilize health education should be encouraged.

We recognize several limitations of our study. First, selection bias may have influenced the results of this study because the participants were a convenient sample of MSM who live in Shenyang. Results from this study may not be applicable to the greater MSM population of China. Secondly, the survey contained questions about sensitive topics, and participants may have felt uncomfortable answering them completely honestly. To minimize such bias, we asked volunteers from local NGOs to act as the interviewers for the study and provided special training for the purposes of this study. Finally, placing the survey location in a hospital might have encouraged PITC acceptance among our study population, creating a social desirability bias. 

Despite these limitations, our study provides valuable insight and lessons regarding PITC acceptance among Chinese high-risk MSM. Our results suggest that strengthening health education on the Internet and improving the utilization of VCTs may increase the willingness to be tested for HIV among Chinese MSM.

## 5. Conclusions 

Our study found that 53.7% of the participants reported a willingness to accept PITC. Additionally, MSM who received VCT more than three times, obtained HIV/AIDS knowledge from brochures or the Internet, and were highly aware of their own HIV risk were most willing to accept PITC. Our results indicate that much greater efforts are needed to lower the barriers to PITC acceptance, improve HIV/AIDS and PITC knowledge, and promote the extension of HIV testing in order to, consequently, improve PITC acceptance among MSM in China, particularly among groups engaged in risky behavior and have a high HIV occurrence.

## Figures and Tables

**Table 1:** Sociodemographic and behavioral characteristics of MSM (*n* = 438).

Demographic/behavioral characteristics	Number of baseline MSM (%, Proportion)
Hometown	
Liaoning	322 (73.5)
Others	116 (26.5)
Age (years)	
Younger (<28)	232 (53.0)
Older (*⩾*28)	206 (47.0)
Ethnic	
Han	370 (84.5)
Others	67 (15.3)
Education	
At least high school	250 (57.1)
Less than high school	188 (42.9)
Occupation	
Unemployed	287 (65.5)
Employed	148 (33.8)
Monthly income (RMB)	
*⩾*1000	289 (66.0)
<1000	149 (34.0)
Looking for sexual partners place	
Internet	205 (46.8)
Park	133 (30.4)
Others	93 (21.2)
Had male regular sexual partner in past 12 months	
Yes	296 (67.6)
No	142 (32.4)
Consistent condom use with regular male sexual partner	
Yes	73 (16.7)
No answer	145 (33.1)
No	220 (50.2)
Had male casual sexual partner in past 12 months	
Yes	352 (80.4)
No	86 (19.6)
Consistent condom use with casual male sexual partner	
Yes	101 (23.1)
No answer	88 (20.1)
No	249 (56.8)

**Table 2:** Willingness for accepting PITC among Shenyang MSM by demographic and sexual behavioral characteristics (unadjusted OR).

Variables	Response	Acceptance (rate, %)	OR	95% CI	*P* value
Age (years)	Younger (<28)	133 (57.3)	0.64	0.30–1.35	0.243
	Older (≥28)	102 (49.5)	1		
Education	At least high school	142 (56.8)	1.34	0.92–1.96	0.128
	Less than high school	93 (49.5)	1		
Occupation	Unemployed	147 (51.2)	1.29	0.86–1.92	0.219
	Employed	85 (56.3)	1		
Monthly income (RMB)	≥1000	150 (51.9)	0.81	0.55–1.21	0.307
	<1000	85 (57.0)	1		
Looking for sexual partners place	Internet	103 (50.2)	0.76	0.47–1.25	0.281
	Park	77 (57.9)	1.04	0.61–1.77	0.892
	Others	53 (53.0)	1		
AIDS prevention knowledge scores	10-11	112 (59.9)	1.55	1.06–2.28	0.024
	<10	123 (49.0)	1		
Previous times of HIV testing within VCT	>3	88 (42.5)	2.91	1.43–5.92	0.003
	1–3	131 (67.9)	1.02	0.51–2.05	0.963
	None tested before	16 (42.1)	1		
PITC benefited oneself	Yes	182 (53.1)	0.9	0.57–1.42	0.638
	No	53 (55.8)	1		
PITC benefited families and friends	Yes	165 (61.1)	2.2	1.49–3.26	<0.001
	No	70 (41.7)	1		
Getting AIDS prevention knowledge from radio	Yes	138 (64.8)	2.43	1.65–3.57	<0.001
	No	97 (43.3)	1		
Getting AIDS prevention knowledge from TV	Yes	167 (59.9)	2	1.34–2.96	0.001
	No	68 (43.0)	1		
Getting AIDS prevention knowledge from newspaper	Yes	170 (61.6)	2.39	1.61–3.56	<0.001
	No	65 (40.4)	1		
Getting AIDS prevention knowledge from friends	Yes	164 (57.1)	1.5	1.01–2.23	0.044
	No	71 (47.3)	1		
Getting AIDS prevention knowledge from leaflet	Yes	167 (62.8)	2.58	1.74–3.83	<0.001
	No	68 (39.8)	1		
Getting AIDS prevention knowledge from Internet	Yes	171 (58.8)	1.85	1.24–2.76	0.003
	No	64 (43.8)	1		
Self-identity high risk of HIV	Yes	36 (72.0)	2.44	1.28–4.67	0.007
	No	199 (51.3)	1		
Self-identity high risk of STD	Yes	194 (55.9)	1.55	0.97–2.46	0.066
	No	41 (45.1)	1		

*PITC: provider-initiated HIV testing and counseling; VCT: HIV voluntary counseling and testing; OR: adjusted odds ratio.

**Table 3:** Willingness for PITC. Multivariate analysis.

Variables	Response	OR (95% CI)	*P* value
Previous HIV testing times within VCT	>3	2.95 (1.36–6.37)	0.006
	1–3	0.94 (0.44–2.02)	0.866
	None tested before		
PITC brought benefit to family and friends	Yes	1.91 (1.25–2.92)	0.003
	No		
Gained AIDS prevention knowledge from brochures	Yes	2.52 (1.64–3.87)	<0.001
	No		
Gained AIDS prevention knowledge from Internet	Yes	1.66 (1.07–2.58)	0.025
	No		
Self-identity high risk of HIV	Yes	2.84 (1.37–5.91)	0.005
	No		

*PITC: provider-initiated HIV testing and counseling; VCT: HIV voluntary counseling and testing; OR: adjusted odds ratio.
